# Supplementary material for: Advanced Glycation End Products Impair Cardiac Atrial Appendage Stem Cells Properties
Source: J Clin Med. 2021 Jul 1;10(13):2964. doi: 10.3390/jcm10132964 (PMC8269351; doi:10.3390/jcm10132964)
Supplement: Supplementary file 1 [file jcm-10-02964-s001.zip › jcm-1264393-supplementary.pdf]

Journal of
Clinical Medicine

MDPI

Supplementary material

Advanced glycation end products impair cardiac atrial appendage stem cells properties

Lize Evens[1], Ellen Heeren[1], Jean-Luc Rummens[1,2], Annelies Bronckaers[1], Marc Hendrikx[1], Dorien Deluyker[1,†], Virginie Bito[1,*,†]

1. Supplementary figures

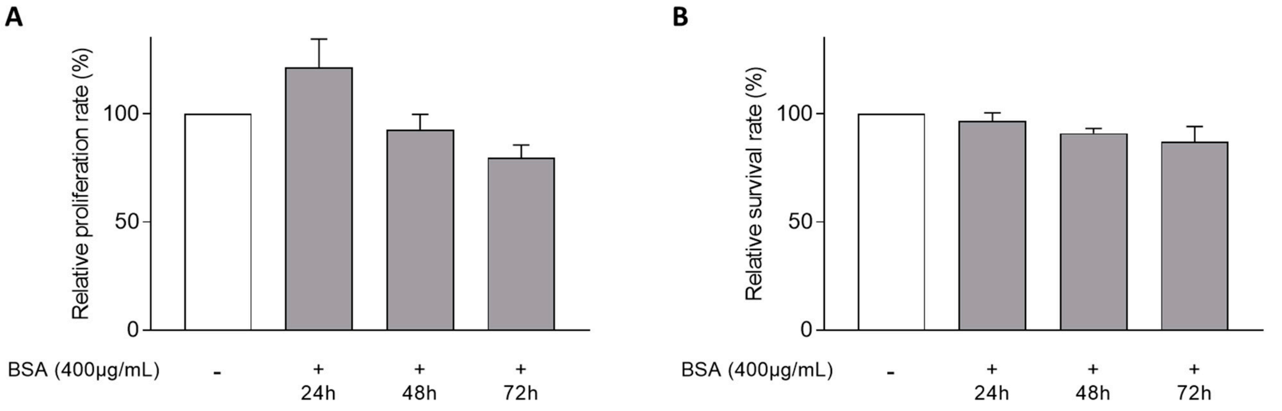

Figure S1. Effect of BSA over time on CASCs proliferation and survival. CASCs were exposed to 400µg/mL BSA for 24, 48, or 72 hours and proliferation (A, n=6) or survival (B, n=7) were evaluated. Data are normalized to the control (media with 2% FCS for proliferation and 0% FCS for survival, both without BSA) and are represented as mean ± SEM.

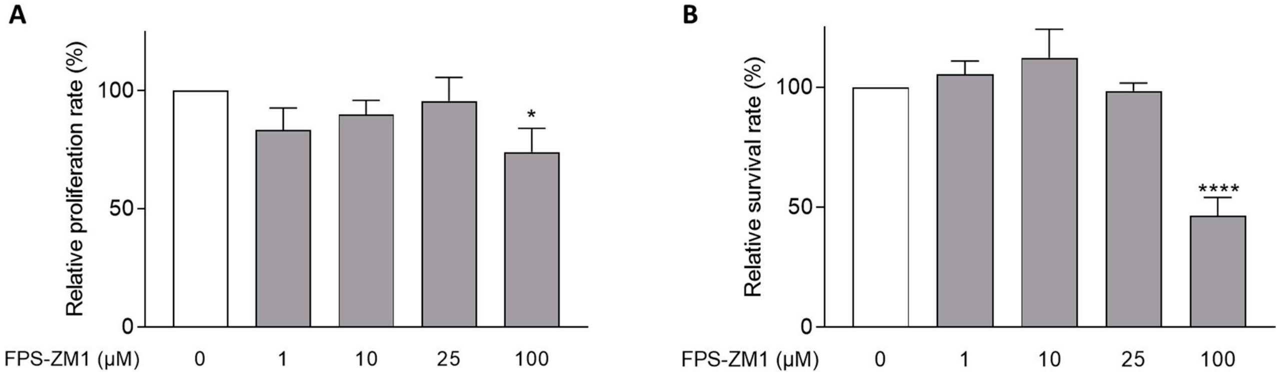

Figure S2. Effect of ranging concentrations of the RAGE inhibitor (FPS-ZM1) on CASCs proliferation and survival. CASCs were exposed to ranging concentrations (0, 1, 10, 25, and 100µM) of FPS-ZM1 for 72 hours in a proliferation (A, n=6) and survival (B, n=5) assay. Data are normalized to the 0µM concentration and are represented as mean ± SEM. *denotes $p<0.05$, and ****denotes $p<0.0001$ *vs.* 0µM FPS-ZM1.
